# Suture Bridge Fixation for Ulnar Sublime Tubercle Avulsion Fractures in High School Baseball Players: A Report of Two Cases

**DOI:** 10.7759/cureus.101770

**Published:** 2026-01-18

**Authors:** Takahiro Kaga, Kenichi Otoshi, Kinshi Kato, Hironori Numazaki, Yoshihiro Matsumoto

**Affiliations:** 1 Department of Sports Medicine, Fukushima Medical University, Fukushima, JPN; 2 Department of Orthopaedic Surgery, Fukushima Medical University, School of Medicine, Fukushima, JPN; 3 Department of Sports Medicine, Fukushima Medical University, School of Medicine, Fukushima, JPN

**Keywords:** adolescent athlete, avulsion fracture, baseball pitcher, medial ulnar collateral ligament, overhead throwing sports, suture bridge fixation, ulnar sublime tubercle

## Abstract

Ulnar sublime tubercle avulsion fractures caused by repetitive valgus stress are rare injuries predominantly observed in adolescent overhead throwing athletes. Although conservative management is typically the first-line treatment, surgical intervention may be required in cases of nonunion, displaced fragments, or persistent medial elbow pain. We report two high school baseball pitchers with symptomatic ulnar sublime tubercle avulsion fractures that were unresponsive to conservative treatment and were surgically treated using a suture bridge fixation technique. In both cases, the small, avulsed fragment was secured with a proximally placed suture anchor, and the suture limbs were tensioned laterally and fixed distally using a knotless anchor to achieve anatomical reduction. Radiographic bone union was confirmed at 16 and 24 weeks postoperatively, after which a progressive throwing program was initiated. Both athletes returned to full competitive pitching at 30 and 28 weeks postoperatively, respectively, without recurrence of symptoms. These cases suggest that suture bridge fixation may be a viable treatment option for adolescent athletes with ulnar sublime tubercle avulsion fractures, particularly when fragment size limits the use of screw fixation.

## Introduction

Ulnar sublime tubercle avulsion fractures are rare injuries predominantly observed in overhead throwing athletes [1-5]. This fracture involves the distal bony insertion of the anterior bundle of the medial ulnar collateral ligament (MUCL) and is thought to result from repetitive valgus overload on the medial elbow during throwing [1,3,4]. Because the anterior bundle of the MUCL has a relatively small and discrete bony footprint on the ulnar sublime tubercle, stable compression at this site is critical for achieving secure fixation. Prior studies have shown that medial epicondyle avulsion fractures, which involve the proximal MUCL attachment, occur at a mean age of 13.1 years [6], whereas sublime tubercle avulsion fractures involving the distal MUCL attachment occur at a mean age of 16.9 years [4]. This age corresponds to a period in which the physis is typically closed or nearing closure, suggesting that sublime tubercle avulsion fractures tend to arise during the later stages of skeletal maturation. Conservative management, including bracing [4], cast immobilization [2], and extracorporeal shock wave therapy (ESWT) [5], is commonly employed as the initial treatment. Surgical intervention may be required for nonunion, displaced fractures, or persistent medial elbow pain despite conservative management. Open reduction and internal fixation using a screw [1] or suture anchors [4] has been described, whereas MUCL reconstruction is reserved for irreparable fragments or cases of ligament insufficiency [7]. The suture bridge technique, originally developed for rotator cuff repair [8], has been adapted for fixation of small avulsion fragments, particularly at ligament or tendon insertions [9]. This transosseous-equivalent construct tensions suture limbs from medial anchors and secures them laterally with knotless anchors, thereby increasing stability and the tendon-bone contact area [8,9]. Giles et al. demonstrated its biomechanical advantages for bony Bankart lesions, showing reduced fragment migration and greater load-bearing capacity compared with single-row anchor repair or direct fragment suturing [7]. These findings suggest that suture bridge fixation may be broadly applicable to small bony avulsion injuries beyond the shoulder. In this study, we report two adolescent baseball players with ulnar sublime tubercle avulsion fractures treated with suture bridge fixation.

## Case presentation

Case 1

A 16-year-old right-handed high school baseball pitcher presented with medial right elbow pain during throwing. He was initially evaluated at a local orthopedic clinic one month after symptom onset. After one month of conservative treatment, including rest, rehabilitation, and extracorporeal shock wave therapy (ESWT), follow-up radiographs were obtained prior to return to throwing; however, no substantial interval change compared with the initial imaging was observed, and definitive fracture healing was not confirmed. Despite the lack of radiographic healing, he returned to sports based on symptomatic improvement and remained asymptomatic while pitching for the subsequent five months. However, five months after resuming pitching, he developed recurrent medial elbow pain and was subsequently referred to our institution.

**Figure 1:**
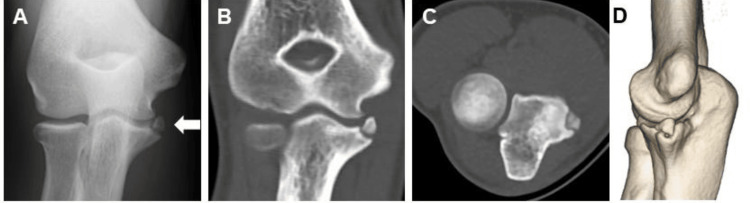
Preoperative radiographic and CT findings of case 1 A. Initial anteroposterior radiograph showing a small bony fragment at the ulnar sublime tubercle (white arrow). B. Coronal CT image. C. Axial CT image. D. Three-dimensional CT reconstruction.

Physical examination revealed localized tenderness over the ulnar sublime tubercle and medial elbow pain with valgus stress. No mechanical symptoms, such as clicking or locking, were observed during the examination. Ultrasonographic evaluation under valgus stress demonstrated no medial joint space widening greater than 2 mm compared with the contralateral side, indicating the absence of gross valgus instability. Plain radiographs demonstrated a 7-mm displaced bony fragment at the ulnar sublime tubercle (Figure 1A), and computed tomography (CT) confirmed the morphology and displacement of the fragment (Figures 1B-1D). The physeal plate was closed on plain radiographs, indicating skeletal maturity. Given the chronic nature of the injury, failure of prior conservative management, and the patient’s desire for an expedited return to competitive pitching, surgical intervention was recommended.

Surgical procedure

**Figure 2:**
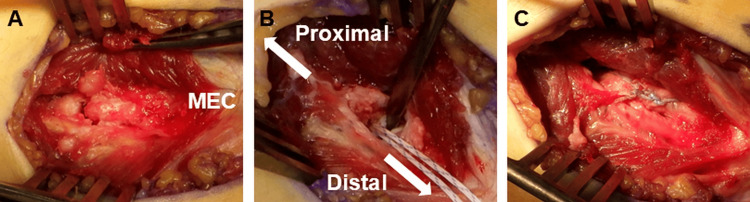
Intraoperative views of case 1 A. Muscle-splitting approach exposing the avulsed bone fragment. B. A soft suture anchor is inserted into the osteochondral transition zone of the footprint. C. Final appearance after completion of the suture bridge construct.

Under general anesthesia, a standard medial elbow approach was used. A longitudinal split was made in the flexor carpi ulnaris (FCU), followed by splitting of the flexor digitorum superficialis (FDS), to expose the anterior bundle of the medial ulnar collateral ligament (MUCL) (Figure 2A). The ulnar nerve was identified and protected without routine retraction. Sharp dissection around the avulsed fragment was performed to preserve its continuity with the MUCL. The fragment was gently reflected, and the bony footprint was debrided to promote osteointegration.

A 1.4-mm ICONIX® suture anchor (Stryker, Kalamazoo, MI, USA) was inserted proximal to the original footprint (Figure 2B). The anchor was seated to the manufacturer-recommended depth using the depth marker on the anchor, with the drill hole oriented away from the joint surface to avoid intra-articular penetration. The suture limbs were passed in a horizontal mattress configuration through the MUCL fibers just proximal to the fragment. To complete the suture bridge construct, the sutures were tensioned laterally and secured using a 3.9-mm SwiveLock® knotless anchor (Arthrex, Naples, FL, USA) placed distal to the sublime tubercle, thereby achieving anatomical reduction of the fragment (Figure 2C). The wound was then closed in layers.

Postoperative course

The elbow was immobilized in a cast for four weeks, followed by a posterior splint for an additional four weeks to minimize valgus stress until early bony healing. Gentle active range-of-motion exercises were initiated several times daily during the splinting period. After splint removal, a valgus-limiting hinged elbow brace was applied for four weeks while a structured rehabilitation program was initiated under the supervision of a physical therapist. The brace was locked to restrict valgus stress, and no specific limits were imposed on elbow flexion or extension. Rehabilitation included progressive passive and active-assisted range-of-motion exercises, along with early kinetic-chain training focusing on the lower extremities and trunk to support proper pitching mechanics.

**Figure 3:**
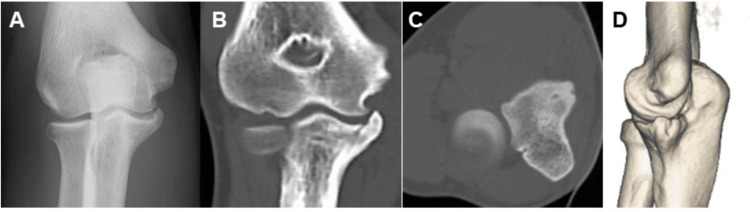
Postoperative radiographic and CT findings of case 1 A. Postoperative anteroposterior radiograph showing satisfactory realignment of the fragment following suture bridge fixation. B. Coronal CT image. D. Three-dimensional CT reconstruction demonstrating successful bone union.

Radiographic bone union was confirmed at 16 weeks postoperatively (Figure 3), after which a gradual throwing program was initiated. The program began with light net throwing during the first week, followed by progressive long toss up to approximately half the regulation throwing distance during the second week. Throwing intensity gradually increased to approximately 70-80% before advancing to full long toss over the subsequent week. Once the patient was able to throw at this intensity without symptoms, bullpen pitching was initiated. Because this was our first experience with this fixation technique, progression through the long-toss phase was intentionally conservative.

The patient returned to competitive pitching at 30 weeks postoperatively without recurrence of symptoms. Objective measurement confirmed that his fastball velocity had returned to the pre-injury level (140 km/h). Near-full pitching intensity was achieved approximately four weeks after initiation of bullpen training, and full recovery was confirmed at 36 weeks.

A 16-year-old left-handed high school baseball pitcher presented with medial left elbow pain during throwing. He had initially undergone one month of conservative management, including rest, rehabilitation, and extracorporeal shock wave therapy (ESWT), at a local orthopedic clinic. After returning to sports, he experienced recurrent medial elbow pain five months later and was subsequently referred to our institution.

**Figure 4:**
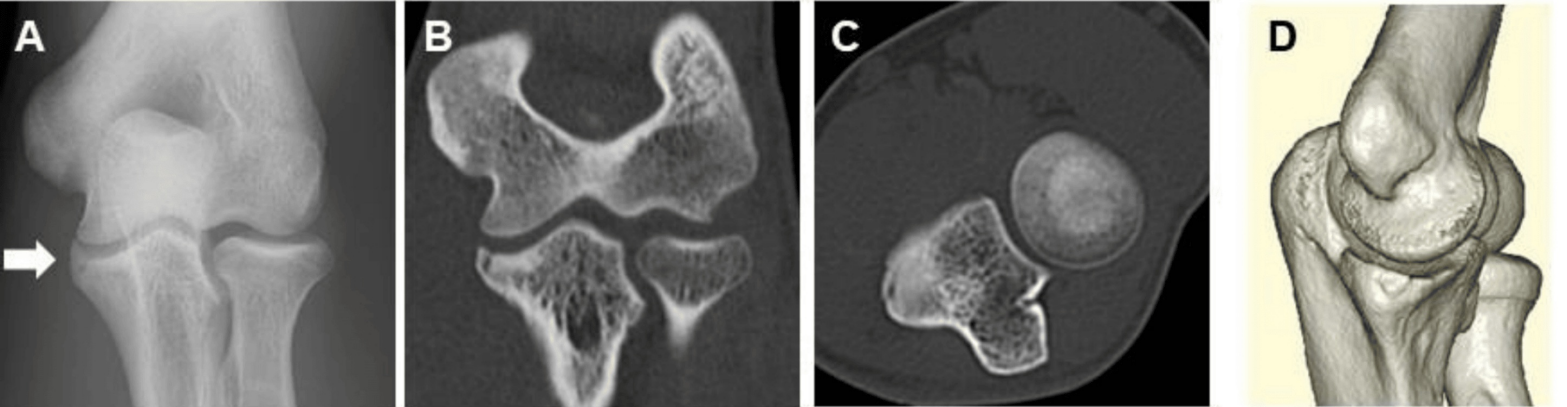
Preoperative radiographic and CT findings of case 2 A. Initial anteroposterior radiograph showing a small bony fragment at the ulnar sublime tubercle (white arrow). B. Coronal CT image. C. Axial CT image. D. Three-dimensional CT reconstruction.

Physical examination revealed localized tenderness over the ulnar sublime tubercle and medial elbow pain with valgus stress. No mechanical symptoms, such as clicking or locking, were observed during the examination. Ultrasonographic evaluation under valgus stress demonstrated no medial joint space widening greater than 2 mm compared with the contralateral side, indicating the absence of gross valgus instability. Plain radiographs demonstrated a 9-mm avulsion fragment at the ulnar sublime tubercle (Figure 4A), and computed tomography (CT) confirmed its morphology and displacement (Figures 4B-4D). The physeal plate was closed on plain radiographs, indicating skeletal maturity. Given the chronic course of symptoms, failure of prior conservative therapy, and the patient’s desire for a prompt return to competitive pitching, surgical treatment was recommended.

Surgical procedure

**Figure 5:**
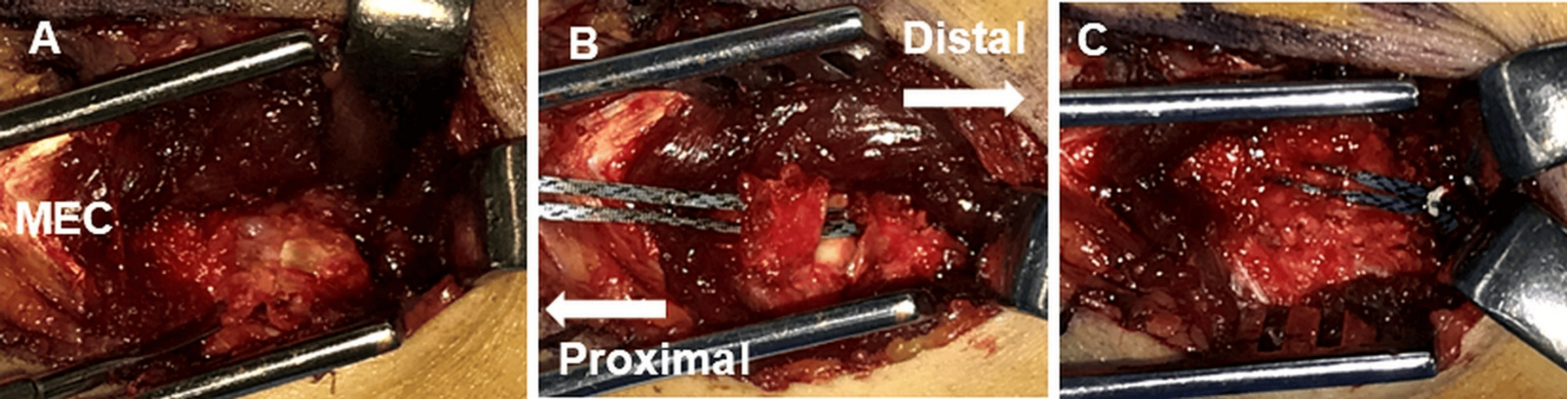
Intraoperative views of case 2 A. Muscle-splitting approach exposing the avulsed bone fragment. B. A soft suture anchor is inserted into the osteochondral transition zone of the footprint. C. Final appearance after completion of the suture bridge construct.

The procedure was performed using the same surgical approach and technique as described for case 1. A 1.4-mm JuggerKnot® soft anchor with tape (Zimmer Biomet, Warsaw, IN, USA) was used instead of the ICONIX® suture anchor (Figure 5). The tape-type anchor was selected to enhance surface contact and distribute compressive forces more evenly across the small avulsion fragment, allowing stable fixation while minimizing the risk of fragmentation. As in case 1, the anchor was seated to the manufacturer-recommended depth with the drill hole oriented away from the joint surface to avoid intra-articular penetration.

Postoperative course

The elbow was immobilized in a posterior splint for six weeks. Gentle active range-of-motion exercises were initiated several times daily beginning in the fourth postoperative week to minimize joint stiffness. After splint removal, a valgus-limiting hinged elbow brace was applied for an additional four weeks while a structured rehabilitation program was initiated under the supervision of a physical therapist. The brace was locked to restrict valgus stress, and no specific limits were imposed on elbow flexion or extension. Rehabilitation included progressive passive and active-assisted range-of-motion exercises, along with early kinetic-chain training emphasizing the lower extremities and trunk.

**Figure 6:**
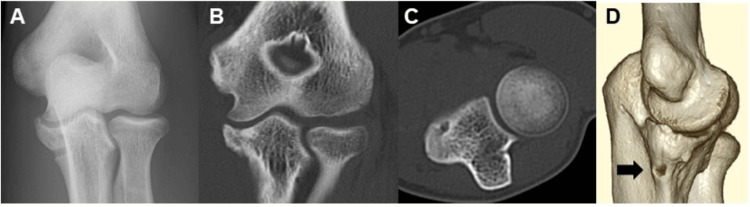
Postoperative radiographic and CT findings of case 2 A. Postoperative anteroposterior radiograph showing anatomical realignment of the fragment following suture bridge fixation. B. Coronal CT image. C. Axial CT image. D. Three-dimensional CT reconstruction demonstrating successful bone union and the position of the lateral knotless anchor used in the suture bridge construct (black arrow).

Radiographic bone union was confirmed at 24 weeks postoperatively (Figure 6), after which a gradual return-to-throwing program was initiated. The postoperative throwing progression followed the same staged protocol as in case 1, including stepwise advancement from net throwing to long toss and subsequent bullpen pitching. The patient progressed smoothly through each phase and resumed full competitive pitching at 28 weeks postoperatively without recurrence of symptoms. At the final follow-up, the patient subjectively reported that his pitching velocity had returned to the pre-injury level, although objective velocity measurements were not obtained. Full recovery was achieved in 30 weeks (Table 1).

**Table 1:** Demographic data, fragment characteristics, and postoperative outcomes of the two cases

Variable	Case 1	Case 2
Age (years)	16	16
Hand dominance	Right-handed	Left-handed
Sport / Position	Baseball / Pitcher	Baseball / Pitcher
Affected side	Right elbow	Left elbow
Fragment size (mm)	7	9
Anchor configuration	ICONIX® suture anchor + SwiveLock® (suture bridge)	JuggerKnot® soft anchor with tape + SwiveLock® (suture bridge)
Reason for anchor selection	Standard suture anchor fixation	Tape-type anchor selected to enhance surface contact and distribute compressive forces
Time to radiographic union (weeks)	16	24
Post-union throwing program	Staged progression (net throwing → long-toss → bullpen)	Same as Case 1
Time to bullpen pitching (weeks)	32	26
Time to return to competitive pitching (weeks)	30	28
Fastball velocity (preoperative)	140 km/h (objectively measured)	Not objectively measured
Fastball velocity (postoperative, final follow-up)	140 km/h (objectively measured)	Subjectively reported as unchanged
Recurrence of symptoms	None	None

## Discussion

Surgical fixation of ulnar sublime tubercle avulsion fractures has been performed using either small screws or suture anchors. Although screw fixation can provide rigid stability, it is generally unsuitable for small avulsion fragments because of the risk of fragmentation or insufficient screw purchase. Accordingly, small avulsion fragments represent a primary indication for anchor-based fixation rather than screw-based open reduction and internal fixation. Historically, one or two anchors have been inserted into the fracture bed with transosseous bone tunnels created in the fragment for horizontal mattress suturing; however, this technique carries a risk of fragment fracture during tunnel creation or suture tightening. An alternative construct occasionally used in clinical practice involves placing anchors into the fracture bed and securing the fragment by wrapping sutures around it in a manner similar to a single-row rotator cuff repair. Compared with such constructs, suture bridge fixation may offer biomechanical advantages by providing broader footprint compression and more uniform contact pressure across the fragment surface, as demonstrated in studies comparing suture bridge and single-row constructs in rotator cuff repair [3,8].

In our cases, the fragment widths (7 and 9 mm) were unsuitable for safe screw fixation, which we consider a specific and primary indication for selecting a suture bridge technique. Based on our experience, avulsion fragments smaller than approximately 10 mm are at increased risk of fragmentation or inadequate screw purchase, making screw fixation potentially unsafe in this setting. Compared with medial ulnar collateral ligament (MUCL) reconstruction, which typically requires at least 12 months before athletes can return to throwing [10], suture bridge fixation may allow earlier functional recovery when fragment size permits secure anchor placement. This technique avoids graft harvesting and preserves native ligamentous anatomy, making it a less invasive option for skeletally immature throwing athletes. However, because the suture bridge construct can generate substantial compressive force, particularly in small avulsion fragments, careful tension control is essential. In our technique, the sutures were deliberately passed over the fragment via the attached ligament to ensure effective compression without loss of fixation, and tape-type sutures were used when appropriate to enhance surface contact and distribute compressive forces more evenly across the fragment.

Despite these advantages, successful fixation requires a sufficiently sized, non-comminuted fragment capable of supporting anchor placement. Extensive displacement, comminution, or combined ligament insufficiency may necessitate primary MUCL reconstruction rather than fragment fixation. Therefore, careful preoperative assessment of fragment morphology, displacement, and ligament integrity is essential when determining the optimal surgical strategy.

A technical challenge associated with this approach is the risk of drill-bit slippage during anchor-hole preparation along the narrow and curved surface of the distal ridge of the sublime tubercle. Precise control of drill trajectory and entry angle is required to avoid misdirection or inadequate penetration. In addition, meticulous anatomical reduction and preservation of surrounding soft tissue are critical for achieving stable fixation and promoting biological healing.

Because sublime tubercle avulsion fractures may be overlooked in adolescent throwers and can mimic traction apophysitis or medial epicondyle pathology, heightened clinical awareness is necessary for timely diagnosis. Early identification of a displaced avulsion fragment allows surgeons to consider fixation strategies that restore native valgus stability without resorting to reconstruction.

Postoperative rehabilitation in our cases emphasized restoration of range of motion, minimization of stiffness, and early kinetic-chain activation, which may reduce compensatory stress on the elbow and facilitate a safe return to throwing. Both athletes returned to competitive pitching within approximately seven months postoperatively without recurrence of symptoms or complications.

Although this report includes only two cases, sublime tubercle avulsion fractures remain rare, and the existing literature consists largely of isolated case reports. In this context, even small case series can provide meaningful clinical insights into fixation strategies for small avulsion fragments in adolescent throwers. While further biomechanical evaluation and larger multicenter studies are required to validate indications, reproducibility, and long-term outcomes, the favorable short-term recovery observed in these cases supports the clinical utility of suture bridge fixation in appropriately selected patients.

## Conclusions

These cases suggest that suture bridge fixation provided stable fixation and successful union in these patients and may represent a viable option for treating ulnar sublime tubercle avulsion fractures in adolescent throwing athletes. The favorable short-term outcomes observed here support its consideration in appropriately selected patients. Sublime tubercle avulsion fractures remain underrecognized in adolescent overhead athletes, and early diagnosis is critical to prevent chronic valgus instability and prolonged time away from sport. Suture bridge fixation may provide a biomechanically stable construct in cases where conservative treatment fails, particularly in high-demand throwers who require early restoration of medial elbow stability. In particular, this technique may be well suited for small avulsion fragments in skeletally mature or near-mature adolescents, in whom screw fixation may be unsafe, and ligament reconstruction may be unnecessarily invasive. Further accumulation of cases and multicenter studies will be necessary to establish optimal surgical indications and to validate the long-term clinical outcomes of this technique.
